# Short term e-bicycle riding results in favorable cardiometabolic shifts in moderately active adults

**DOI:** 10.1007/s00421-024-05418-1

**Published:** 2024-02-01

**Authors:** Helaine M. Alessio, Kevin D. Ballard, Paul T. Reidy, Katie M. Hayward, Alexandra M. Bagg, Rachel A. Cooley, Michael J. O’Connell, Alexander H. K. Montoye, Kyle L. Timmerman

**Affiliations:** 1 Department of Kinesiology, Nutrition, and Health, Miami University, Oxford, USA; 2 Department of Statistics, Miami University, Oxford, USA; 3 Integrative Physiology and Health Science Department, Alma College, Alma, USA

**Keywords:** EB, Pedelec, Continuous glucose monitoring, Central arterial stiffness

## Abstract

**Purpose:**

Electric bikes (EB) are a form of active transportation with demonstrated health benefits. The purpose of this study was to determine the influence of riding an EB for one week on indices of cardiometabolic health in middle-aged adults.

**Methods:**

Adults (n = 22; age = 57.1 ± 11.3 year; BMI = 27.7 ± 4.9) participated in a 2 week study. During Week 1, participants were instructed to continue regular activities. Starting Week 2 participants were provided an EB to ride at least 3 days for a minimum of 30 min·day^−1^. Physical activity (PA) and glucose were measured continuously. Body composition, blood lipids, glucose, insulin, hemoglobin A1c (HbA1c), plasma endothelin-1 (ET-1), and carotid-femoral pulse wave velocity (cf-PWV) were measured on days 1 and 14.Data and Statistical analyses or Statistics. Each participant served as their own control. Paired t-tests compared dependent variables between week 1 (without EB) and week 2 (with EB).

**Results:**

When provided an EB for one week, moderate to vigorous PA increased by 6–9 min·day^−1^ (P < 0.05) and sedentary time decreased by ~ 77 min·day^−1^ (P < 0.05). Data from 24 h continuous glucose monitoring showed the percentage of time in healthy range (70–120 mg·dl^−1^ glucose) increased (P < 0.05) from week 1 to week 2. Compared to day 1, cf-PWV was lower at day 14 (P < 0.05) following one week of riding an EB.

**Conclusion:**

Moderately-active, middleaged adults showed improved continuous glucose regulation and lower central arterial stiffness following one week of riding an EB.

## Introduction

The health benefits associated with regular physical activity for reducing morbidity and mortality are well documented (Warburton and Bredin 2017; Ruegsegger and Booth 2018). However, much of the world’s population is not meeting minimal physical activity guidelines (Guthold et al. 2018). Persuading more people to engage in physical activity is challenging. Commonly reported barriers to engaging in physical activity include lack of time and perception that physical activity is not fun (Withall et al. 2011). Making physical activity more fun, as well as integrating physical activity into activities such as commuting or leisure, may be viable ways to increase physical activity in some populations, especially those whose fitness levels fall below the guidelines for regular physical activity (Lakicevic et al. 2020). Electric bicycle (EB) use has been associated with healthy, fun, and environmentally-friendly options for transportation (Alessio et al. 2021). There is growing interest in investigating glycemic variation as a clinical meaningful indicator for vascular and metabolic complications, especially in middle-aged adults where aging and diabetes risk are directly related (Chia et al. 2018). Thus, the primary objective of this study was to determine the impact of one week of EB use on glycemic variation in middle-aged adults.

EBs were first produced over 100 years ago, however, it has only been in the past decade that they have gained popularity, and in fact, are the fastest growing sector in the bicycle market. EBs represent approximately 15% of all bicycle sales globally, with a market value of $35.7 billion (Balton 2022). An EB is a bicycle equipped with a battery-powered motor that provides an assist when pedaling. A motor increases the speed the rider can travel when pedaling and/or the distance that can be ridden, while typically requiring reduced effort during the ride. The battery capacity on EBs is limited to 20–80 miles. When using lower assist levels, the distance range is maximized while higher assist levels deplete the battery more rapidly, limiting the distance capacity. The assist provided by the battery-powered motor reduces the effort required of riders, making EBs a potentially favorable transportation option for leisure or commuting. A meta-analysis reported that personal use of EBs were associated with active commuting, shopping, visiting family and friends and for leisure recreation (Bourne et al. 2020), yet only 3% of US adults report using active transportation (e.g., cycling or walking) to get to work (Whitfield 2020). In a randomized control study conducted in a real-life setting, EBs with trailers were provided to parents with no specific directions about when to use them (Bjørnarå et al. 2019). Results showed that the EBs were used in place of car trips for daily tasks that included dropping a child off at school, commuting to work, and running errands. A recent study from our laboratory (Alessio et al. 2021) and a recent meta-analysis (McVicar et al. 2022) showed that acute EB riding increased energy expenditure, heart rate, oxygen consumption (VO_2_), and metabolic equivalents to levels indicative of moderate-intensity exercise, suggesting that regular EB use may benefit health and fitness. In our study (Alessio et al. 2021), virtually all participants perceived EB riding as easy and fun even as they rode at moderate intensities with the perception of lower effort.

An analysis of 20 studies reported that EB riding frequency ranged from 2–5 days·week^−1^ and distances ranged from 2.7 to 24.0 km, with the majority reporting a mean daily distance between 3 and 11.5 km (Bourne et al. 2020). Fourteen studies analyzed in a meta-analyses showed significantly increased heart rate, VO_2_ and metabolic response (McVicar et al. 2022) and a study on individuals with different fitness levels reported health benefits associated with EB riding with improved peak oxygen consumption (VO_2_peak) ranging from 8.0 to 9.6% in individuals with low fitness compared with a 1.5% increase in individuals with high fitness (Bourne et al. 2018).

We recruited middle-aged adults who did not regularly ride any type of bicycle with the intent to determine if short-term EB use affected markers of cardiometabolic health. Recent meta-analyses suggest that beneficial changes in continuous glucose profiles (Munan 2020) and arterial pulse wave velocity (PWV) (Saz-Lara 2021) are plausible with short-term (< 2 weeks) physical activity. Thus, the primary objective of this study was to determine the impact of one week of EB use on glucose regulation in middle-aged adults. We hypothesized that short-term EB use would improve glucose regulation as measured by continuous glucose monitors. A secondary objective was to determine the impact of short-term EB use on indices of cardiovascular health. We hypothesized that short-term EB use would improve cardiovascular health as measured by arterial PWV and plasma endothelin-1 (ET-1).

## Methods

### Participants

We conducted a power analysis based on previous literature to determine the necessary sample size to reach 80% power to detect a difference of 10 mg/dL in average glucose. The 10 mg/dL difference is based on the change in average glucose for resting compared to exercise (DuBose et al. 2021), but we used a smaller value because we expected a smaller change based on EB usage. We used a standard deviation of 17 mg/dL (Shah et al. 2019). Based on this analysis, the necessary sample size to reach 80% power was 13 subjects. Twenty-three middle-aged, healthy adults (13 women; age = 52.8 ± 12.7 years (range = 28–67 years) participated in the present study (Table 1). None of the participants indicated that they regularly rode any type of bicycle. They all self-reported walking on average five days each week and engaging in moderate physical activity an average of three days each week. Based on IPAQ data, virtually all participants were active and met the minimum guidelines for physical activity. All participants gave written informed consent for the study after receiving a detailed explanation of the study and study procedures were conducted in accordance with Miami University’s Internal Review Board. All human studies have been approved by the appropriate ethics committee and have therefore been performed in accordance with the ethical standards laid down in the 1964 Declaration of Helsinki.

**Table 1:** Participant characteristics (n = 23)

Variables	Mean ± SD
Age (years)	52.8 ± 12.7
Height (cm)	171.7 ± 10.6
Body mass (kg)	81.7 ± 18.1
Body mass index (kg·m^2^)	27.7 ± 4.9
Body fat (%)	32.5 ± 10.0
Resting systolic blood pressure (mmHg)	122.2 ± 17.5
Resting diastolic blood pressure (mmHg)	77.5 ± 11.2
Resting heart rate (b·min^−1^)	68.5 ± 12.0
Total cholesterol (mg·dl^−1^)	180.8 ± 33.1
HDL-C (mg·dl^−1^)	54.0 ± 16.1
LDL-C (mg·dl^−1^)	115.0 ± 24.3
Triglycerides (mg·dl^−1^)	89.7 ± 53.0
HbA1c (%)	5.3 ± 0.4
Blood glucose (mg·dl^−1^)	100.9 ± 21.0
Plasma insulin (*u*IU·ml^−1^)	7.3 ± 9.4
HOMA-IR	2.9 ± 4.2
Plasma endothelin-1 (pg·ml^−1^)	2.2 ± 0.9

### Study design

This was a crossover study with the first week serving as a control for all. Participants reported to the laboratory at the beginning of week one (day 1) and at the end of week two (day 14). During the first week, participants were instructed to continue their regular activities. On day 7, participants were provided an EB to ride at least 3 days that week, for a minimum of 30 min·day^−1^. Each participant served as their own control. The EB (Gazelle Medeo T9 HMB, Dieren, Netherlands) was a class 1 step-through pedal electric with 20 mph top speed. Power was provided by a Bosch Active Line Plus (50 Nm) engine with 400 Wh connected to a 36 V lithium ion battery. The EB weighed 19 kg with 2.7 kg attributed to the battery and rack. The battery provided power to the bicycle crank during pedaling, which assisted the rider up to a speed of up to 20 mph, a state requirement to be considered a bicycle. The electric system has both speed and impulse sensors that determine the amount of assistance provided by the motor based on the assistance level setting that each participant controls on the handlebar and the force each participant applies on the pedals.

### Study variables

On day 1 and day 14, participants had their height, body composition (InBody 770, Cerritos, CA), resting blood pressure and heart rate (OMRON, Kyoto, Japan), carotid-femoral pulse wave velocity (cf-PWV), blood lipids and glucose, plasma insulin, hemoglobin A1c (HbA1c), and plasma endothelin-1 (ET-1) measured following an overnight fast.

On day 1, participants completed a YMCA submaximal cycle ergometer test to predict VO_2_peak (Golding 2000). This test was performed on a stationary cycle ergometer and lasted between 7 and 12 min. Heart rate (Polar T31 HR monitor (Kempele, Finland) and rating of perceived exertion (RPE) were monitored every minute during the submaximal test. Next, participants had a continuous glucose monitor (FreeStyle Libre Pro, Abbott Laboratories, Chicago, IL) placed on the back of their arm as well as a physical activity monitor (PAL Technologies, Scotland UK) placed on their thigh. Both monitors were worn for the entire 14 day duration of the study. If a sensor fell off the participant was provided a new sensor as soon as possible. Data was extracted and exported as txt files to GlyCulator 3.0 (Chrzanowski et al. 2023) for analysis of 24 h daily averages of mean glucose, glucose standard deviation (SD), glucose coefficient of variation (CV), and percentage of time in healthy range (TIR) (70–120 mg·dl^−1^). Only days with 24 h of complete data were used and 24 averages of the days of week 1 and week 2 were compared. Data from the ActivPAL accelerometers were exported into PAL software suite version 8 for activPAL management, data visualization and data processing, and outcomes were determined using the CREA enhanced algorithm (Montoye et al. 2022); only days with a full 24 h of wear were included in the analysis, and participants had to have at least three days of included ActivPAL data in each week to be included in the analysis. Active outcome measures from the ActivPAL included total daily steps, activity score (METs), upright time, stepping time, cycling time, moderate- and vigorous-intensity time, and breaks in sedentary time. Sedentary outcome measures included primary and secondary lying time, seated transport time, average duration of sedentary bouts and total sedentary time.

Carotid-femoral pulse wave velocity (cf-PWV) was measured on day 1 and day 14 using the SphygmoCor XCEL system (AtCor Medical, Naperville, IL) following 10 min of supine rest. All cf-PWV measurements were performed by the same trained technician. Due to technician availability, baseline and post-intervention cf-PWV values are reported for 16 participants.

Whole blood was collected from an antecubital vein into evacuated tubes for the determination of fasting blood lipids, glucose (Abbott Cholestech LDX Analyzer, Abbott Labs, Orlando, FL), and HbA1c (Alere Afinion, Abbott Labs, Orlando, FL). Plasma was obtained by centrifugation and stored at − 80 °C until analyzed. Plasma endothelin-1 (ET-1) was measured in duplicate (intra-assay CV = 5.1%) as previously described (Ballard et al. 2021) using an enzyme-linked immunosorbent assay (ELISA) kit in accordance with the manufacturer’s instructions (R and D Systems, Minneapolis, MN). Plasma insulin was measured using an ELISA kit in accordance with manufacturer’s instructions (Alpco, Salem, NH). Homeostatic model assessment of insulin resistance (HOMA-IR) was calculated (Gutch et al. 2015).

### Statistical analysis

All descriptive statistics are shown with mean and standard deviations. Paired t-tests were performed to compare the dependent variables between Week 1 (without EB) and Week 2 (with EB). A box plot was used to visualize the difference in pulse wave velocity between Week 1 and Week 2, with profiles to show the change for each individual subject. All statistical analyses were performed with R version 4.1.1 (R Core Team 2020). Normality was assessed with normal quantile–quantile plots, which determined that parametric methods were reasonable.

## Results

**Fig. 1:**
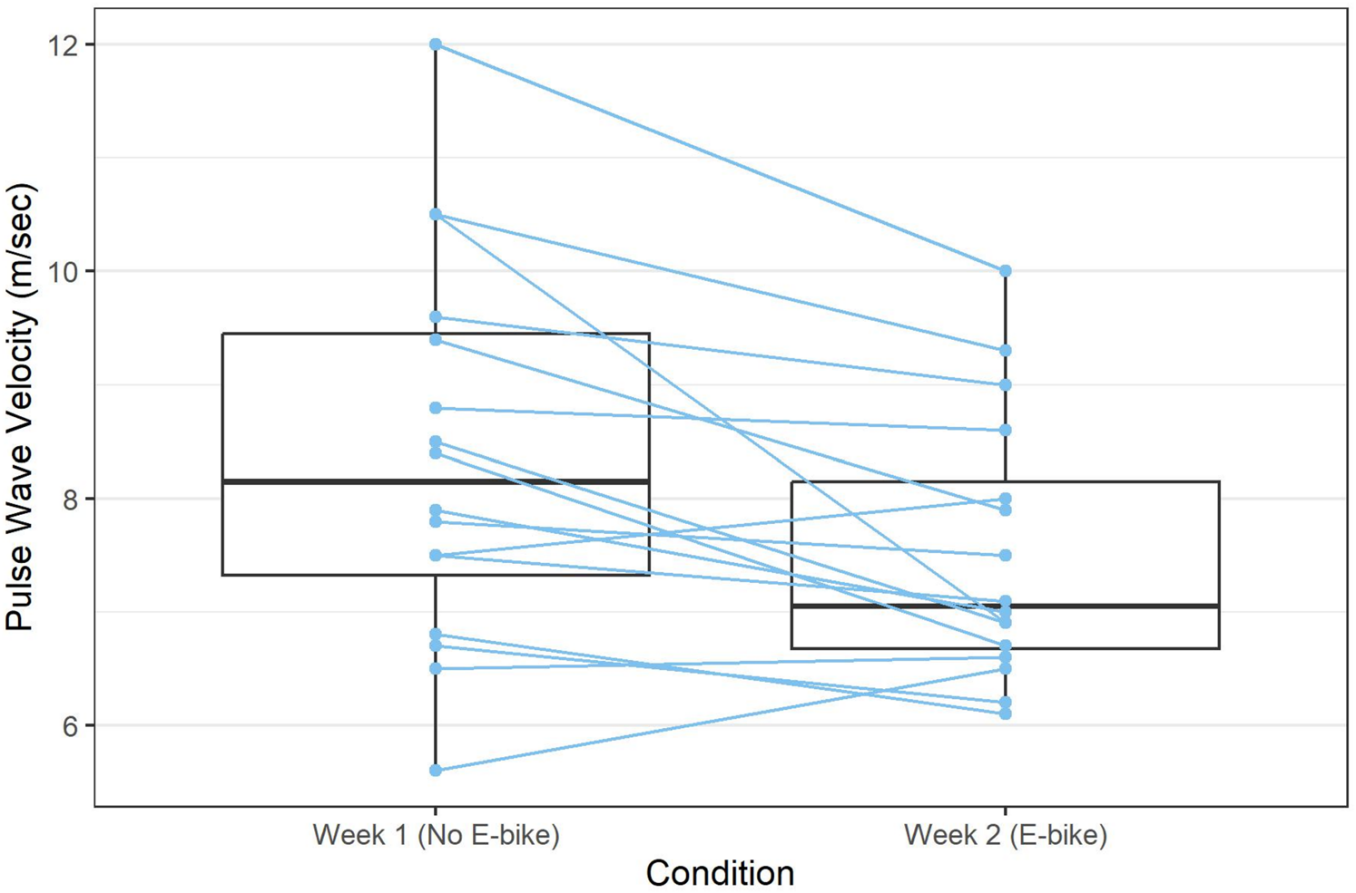
Carotid-femoral pulse wave velocity (cf-PW) on day 1 and day 14. Data (n = 16) are mean ± SD

Participants (Table 1) were 28–67 years of age and overall healthy with no restrictions for physical activity. Participants’ VO_2_peak = (29.2 ± 5.8 ml·kg^−1^·min^−1^) reflected below average to average fitness level for men and women (Liguori et al 2021). No changes were observed from day 1 to day 14 in body composition, resting blood pressure or heart rate, blood lipids, blood glucose, plasma insulin, HOMA-IR, HbA1c, or plasma ET-1 (Table 1). Compared to day 1, cf-PWV was lower at day 14 (P < 0.05) following one week of riding an EB (Fig. 1).

Self-reported physical activity from the IPAQ indicated that virtually all study volunteers engaged in low to moderate-intensity activity 3 days·week^−1^, yet spent more than 70% of a typical work day sitting. Table 2 provides mean and standard deviations for answers to the seven IPAQ questions. Using IPAQ scoring procedures, one individual was categorized as being inactive, 14 individuals were categorized as being minimally active and 8 individuals were categorized as moderately active, engaging in health enhancing physical activity (HEPA) active.

**Table 2:** Baseline responses to seven questions from the International Physical Activity Questionnaire (IPAQ)

IPAQ Questions	Mean ± SD
During the **last 7 days**, on how many days did you do **vigorous** physical activities like heavy lifting, digging, aerobics, or fast bicycling?	0.9 ± 1.5 days
How much time did you usually spend doing **vigorous** physical activities on one of those days?	21.3 ± 43.2 min·day^−1^
During the **last 7 days**, on how many days did you do **moderate** physical activities like carrying light loads, bicycling at a regular pace, or doubles tennis? Do not include walking	3.0 ± 2.7 days
How much time did you usually spend doing **moderate** physical activities on one of those days?	56.8 ± 63.8 min·day^−1^
During the **last 7 days**, on how many days did you **walk** for at least 10 min at a time?	6.1 ± 1.3 days
How much time did you usually spend **walking** on one of those days?	86.6 ± 80.7 min·day^−1^
During the **last 7 days**, how much time did you spend **sitting** on a **week day**?	338.6 ± 164.2 min·day^−1^

Accelerometry data indicated slight differences in moderate physical activity between the two weeks with the major difference in the higher amount of vigorous activity during the EB week. During week 2 when participants were provided an EB, average cycling time increased (P < 0.05) by approximately 12 min·day^−1^ compared to week 1 (Table 3). Additionally, step counts increased (P < 0.05) by approximately 1600 steps·day^−1^, activity score increased (P < 0.05) by ~ 0.02 METs, MVPA increased (P < 0.05) by 6–9 min·day^−1^ and upright time increased (P < 0.05) by 29 min·day^−1^ in week 2 compared to week 1. Sedentary time decreased (P < 0.05) by ~ 77 min·day^−1^ in week 2 compared to week 1 (Table 2), and the total number of breaks in sedentary time decreased (P < 0.05) by an average of nearly four fewer breaks per day in week 2 compared to week 1. However, this difference was no longer statistically significant after adjusting for the duration of sedentary time (i.e., calculating average duration of sedentary bouts). There were no differences in primary lying time (a surrogate measure of sleep) or secondary lying time (a surrogate for daily napping or other lying behaviors), total stepping time, seated transport time, or time spent separately in moderate-intensity physical activity or vigorous-intensity physical activity. When asked in an open-ended survey about the extent to which they enjoyed riding the EB, the majority of responses were positive and indicated they were fun and easy to ride.

**Table 3:** Objective physical activity data

activPAL metrics	Week 1	Week 2
Steps·day^−1^	9406 ± 4035	10,537 ± 3313*
Activity score (average METs across day)	1.43 ± 0.07	1.45 ± 0.1*
Sedentary time (min·day^−1^)	572.9 ± 117.0	491.6 ± 116.0*
Total upright time (min·day^−1^)	368.7 ± 125.6	407.4 ± 124.3*
Total stepping time (min·day^−1^)	117.1 ± 51.1	117.7 ± 41.5
Upright, non-stepping time (total upright–total stepping; min ·day^−1^)	251.6 ± 92.3	289.7 ± 97.1*
Cycling time (min·day^−1^)	2.4 ± 3.9	14.8 ± 10.7*
Primary lying time (surrogate for overnight sleep; min·day^−1^)	462.7 ± 88.7	491.1 ± 100.3
Secondary lying time (surrogate for daily napping or other lying behaviors; min·day^−1^)	35.5 ± 55.1	49.9 ± 50.2
Total lying time (primary + secondary; min·day^−1^)	498.2 ± 84.5	541.0 ± 114.8*
Seated transport time (min·day^−1^)	77.9 ± 41.6	57.7 ± 29.5
MVPA using 3 MET threshold (min·day^−1^)	30.2 ± 21.8	35.1 ± 19.1
MVPA based on stepping cadences of ≥ 100 steps/min (min·day^−1^)	14.5 ± 13.5	18.6 ± 14.7
Moderate-intensity physical activity based on stepping cadences of 100–129 steps/min (min·day^−1^)	13.3 ± 12.6	16.2 ± 12.5
Vigorous-intensity physical activity based on stepping cadences of ≥ 130 steps/min (min·day^−1^)	0.9 ± 2.4	3.8 ± 6.7
Breaks in sedentary time (#·day^−1^)	56 ± 15	52 ± 12*
Average duration of sedentary bouts (min·bout^−1^)	10.9 ± 3.7	9.9 ± 3.0

Data are mean ± SD

*P < 0.05 between Week 1 and Week 2

**Fig. 2:**
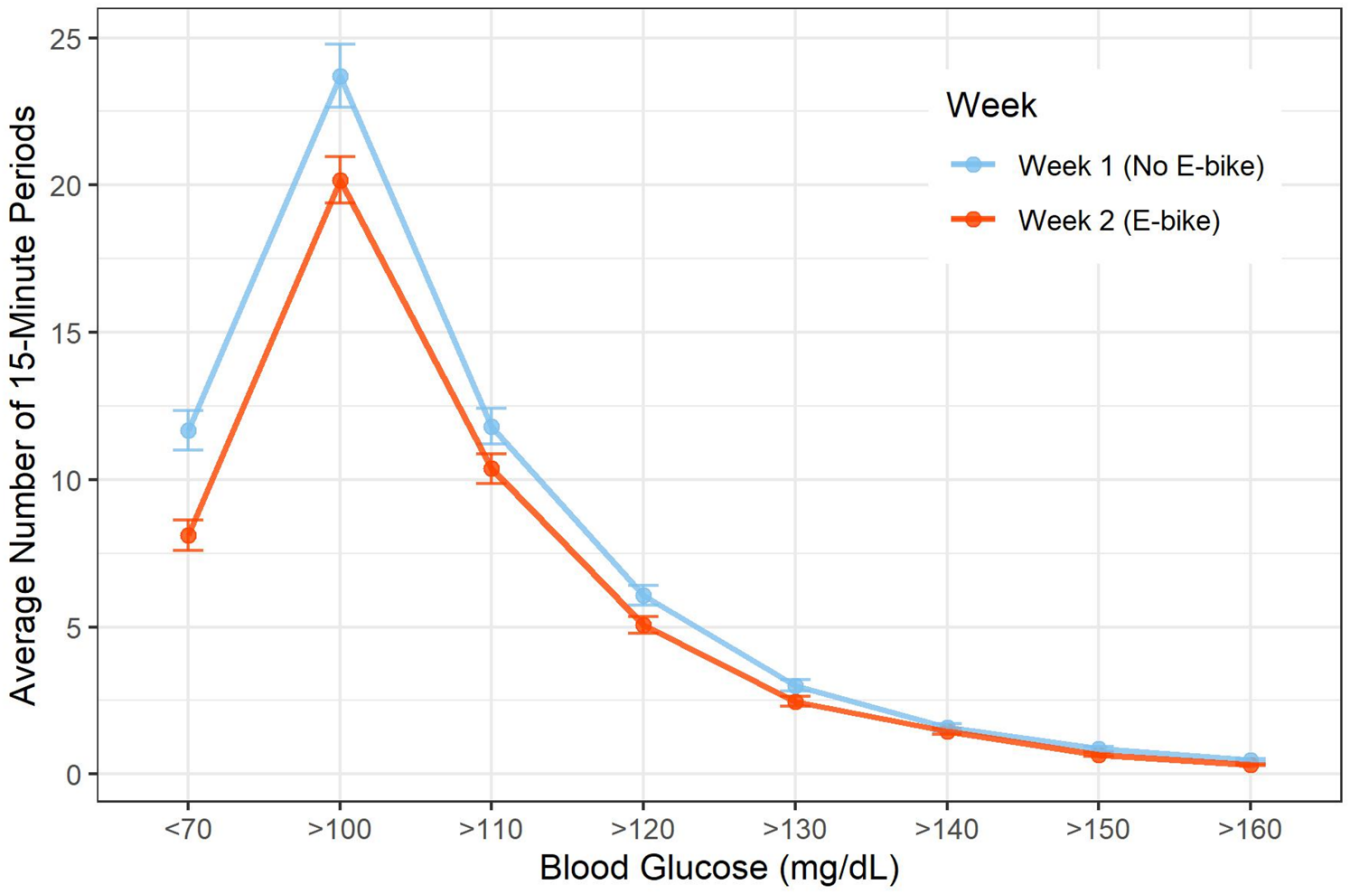
Continuous glucose data showing average 15-min periods throughout the day in which glucose levels cross a specified threshold. The bars show the standard error for each point

Continuous glucose data for week 1 (no EB) and week 2 (with EB) differed when comparing average 15-min periods throughout the day in which glucose levels crossed specific thresholds between 70 and 130 mg·dl^−1^ (Fig. 2). No changes in 24 h mean glucose, 24 h glucose SD, or 24 h glucose CV were observed yet percentage of time in healthy range (TIR) increased from week 1 to week 2 (Table 4).

**Table 4:** Continuous glucose metrics

	Week 1	Week 2	Non-diabetic normative values (mean±SD or range)
24h mean glucose (m·dl^−1^)	87.6 ± 10.4	90.8 ± 8.1	99 ± 6
24h glucose SD (m·dl^−1^)	14.6 ± 2.3	14.3 ± 3.3	16 ± 3
24h glucose CV (%)	16.7 ± 2.7	15.7 ± 3.1	16 ± 3
TIR 70-120 mg·dl^−1^ [%]	81.2 ± 11.2	89.4 ± 11.9*	89 (86–91)

Data are mean±SD or ranges

CGM measurements are taken from averages of 24 hour periods. Time in range (TIR). *P<0.05 between Week 1 and Week 2. Normative values were provided from (Shah et al. 2019).

## Discussion

When provided with an EB for one week, nearly all participants had lower sedentary time and increased daily physical activity, in large part due to riding the EB. We measured resting blood pressure, and one time measures of blood glucose and lipids, as well as body composition mainly to determine if any fluctuations occurred that may be attributed to the EB intervention. No changes in body composition, blood pressure, blood lipids, glucose, and insulin were observed between week 1 without an EB and week 2 with an EB. Body composition and blood pressure are typically difficult to change in a short period of time, especially without weight loss resulting from caloric restriction and regular exercise training (Byambasukh et al. 2021; Cornelissen and Smart 2013; Fagard 2005; Roberts et al. 2002).

Little research exists regarding the effect of EB riding on metabolic health, specifically glucose metabolism and insulin resistance. In one study, EBs provided to sedentary commuters for 4 weeks reduced 2 h blood glucose levels following an OGTT, but not the fasting blood glucose or HOMA-IR (Peterman et al. 2016). We observed a similar lack of change in fasting blood glucose and HOMA-IR. We did observe, using continuing glucose monitors, an increase in the time in healthy range (70–120 mg·dl^−1^), illustrating better glucose control during the EB week. Our CGM data is comparable to normal values for non-diabetic 25–60 years adults (Shah et al. 2019), especially after the EB week. Also, our observed improvements are similar in scale to those reported in type II diabetic adults where glycemic regulation improved following one week of aerobic exercise training (Mikus et al. 2012). In that study (Mikus et al. 2012), postprandial glucose as measured by CGM, improved even though average glucose and insulin responses to an OGTT did not. It is important to note that study participants engaged in aerobic exercise for 60 min·day^−1^ at 75% HRR, whereas our participants increased their physical activity by approximately 12 min·day^−1^ riding an EB as well as an additional 1131 steps·day^−1^ and 38 min·day^−1^ of up-right non-stepping time, and yet still saw some metabolic benefits. A recent study (Smith et al. 2021) showed that a 3 week intervention of interrupted sitting increased physical activity by approximately 10 min·day^−1^), but resulted in subtle changes (16.3 vs. 14.3%, P = 0.04) in the daily standard deviation of glucose when normalized to each individual’s average daily glucose level.

During the first week, accelerometry data recorded an average of 2.4 min of cycling time/day. This is likely not due to cycling but possibly another physical activity that was close to cycling, including high stepping or another similar activity. Day 7 is the end of Week 1, the control week. Data collected at Day 7 represents the control condition in accelerometry and continuous glucose monitoring. At the beginning of week 2 (Day 8), individuals were advised, not required, to ride the EB for at a minimum of 30 min·day^−1^, 3 days·week^−1^. According to accelerometry data the average cycling time was 14.8 min·day^−1^ when averaged across the number of valid days of accelerometer data provided by the participant (average 5.5 days·week^−1^), so it does appear that participants rode the EB close to the total duration suggested by the researchers. Their moderate—to vigorous intensity physical activity increased by as much as 8.9 min·day^−1^ (~ 62 min·week^−1^). Using a ≥ 130 steps·min^−1^ stepping cadence threshold to represent vigorous-intensity physical activity (Ayabe et al. 2011) which would not encompass time spent cycling, there was a non-significant trend (P = 0.06) toward more time spent in vigorous-intensity physical activity in Week 2 compared to Week 1 (Table 3). Nonetheless, the majority of the increased total physical activity in Week 2 is attributed to increased light- and moderate-intensity physical activity completed by participants in the week they had the EB. Additionally, we found that the increased physical activity from riding the EB also decreased their sedentary behavior in the week riding the EB, providing evidence, at least in the short term, that EB use did not result in compensatory increases in sedentary behavior or decreases in moderate- or vigorous-intensity physical activity outside of cycling time across the day or week.

Increased central arterial stiffness is implicated in the development of CVD (Fernberg, Fernström, and Hurtig-Wennlöf 2021). Endothelin-1 (ET-1) is a vasoconstrictor molecule implicated in conditions such as hypertension, congestive heart failure, and inflammation (Li et al. 2020). Plasma ET-1 concentrations are directly related to central PWV, a measure of central arterial stiffness, independent of blood pressure (Otsuki et al. 2007). This association suggests that strategies to decrease plasma ET-1 may improve arterial stiffness, thereby lowering CVD risk. Studies show that moderate- to vigorous-intensity aerobic exercise training decreases plasma ET-1 concentrations (Lewczuk et al. 2003; Maeda et al. 2004; Dow et al. 2017) and central PWV (Stamatelopoulos et al. 2020; Fernberg, Fernström, and Hurtig-Wennlöf 2021). One study found that six consecutive days of prolonged, vigorous-intensity cycling (65% VO_2_peak for 2 h) decreased central PWV by 9% in young men (Currie et al. 2009). To our knowledge, no studies have determined the effects of EB riding on central arterial stiffness and plasma ET-1. Participants in the present study performed little vigorous-intensity physical activity, instead spending the majority of cycling and other movement activities at low- to moderate-intensity levels. Despite our lower intensity intervention compared to others (Currie et al. 2009), we found a 6% decrease in central PWV after one week of EB riding in healthy middle-aged adults, independent of changes in plasma ET-1. The long-term benefits of EB riding on CVD risk factors warrant further investigation.

A second result of interest was the continuous glucose data. Continuous glucose levels reflect the acute impact of exercise on blood glucose patterns. We report data from averaging 15-min periods throughout the day when blood glucose levels crossed a specified threshold, with values of 130–139, 140–149, and 150 + considered as unhealthy ranges. We observed that during the first week without the EB, most blood glucose levels crossed the threshold into unhealthy ranges more often compared with Week 2 when they were riding the EB. The actual number of 15-min periods during the day whereby blood glucose levels rose above 130 mg·dl^−1^ decreased 37.5% when individuals rode the EB in Week 2. This reflects a healthier glycemic regulation when individuals were more active, which supports several exercise studies, although most were of longer duration than one week (Kirwan et al. 2009; Najafipour et al. 2017). The result showing that glycemic variability was impacted significantly within one week while riding an EB, was surprising given the short time riding the EB.

This is a preliminary study that showed there is some promise for improved cardiometabolic health benefits with EB usage. However, the current study design may have some confounding with time, since all study participants used no EB in the first week and an EB in the second week. A future randomized trial can help to control for the temporal effects of the study duration.

## Conclusion

EB riding is gaining popularity globally as more people, regardless of fitness level or age, use EBs for either work, leisure, or running errands. We showed that riding an EB for one week increased physical activity levels, decreased sedentary time, lowered central arterial stiffness, and improved glycemic control in middle-aged adults. Our findings support short-term cardiometabolic benefits of EB riding.

## Data Availability

The data supporting the conclusions of this article can be made available by the authors, upon request.

## References

[CR1] Alessio HM, Reiman T, Kemper B (2021). Metabolic and cardiovascular responses to a simulated commute on an e-bike. Transl J Am Coll Sports Med.

[CR2] Ayabe M, Aoki J, Kumahara H, Tanaka H (2011). Assessment of minute-by-minute stepping rate of physical activity under free-living conditions in female adults. Gait Posture.

[CR3] Ballard KD, Timsina R, Timmerman KL (2021). Influence of time of day and intermittent aerobic exercise on vascular endothelial function and plasma endothelin-1 in healthy adults. Chronobiol Int.

[CR4] Balton J (2022) 101+ Bike Statistics and Facts of 2023 [E-Bikes Included). In: bicycle guide-Bike Rev. Cycl. Advice Best Picks Mt. Road Hybrid Electr. Bikes More. https://www.bicycle-guider.com/bike-facts-stats/. Accessed 27 Mar 2023

[CR5] Bjørnarå HB, Berntsen S, te Velde JS (2019). From cars to bikes—the effect of an intervention providing access to different bike types: a randomized controlled trial. PLoS One.

[CR6] Bourne JE, Cooper AR, Kelly P (2020). The impact of e-cycling on travel behaviour: a scoping review. J Transp Health.

[CR7] Bourne JE, Sauchelli S, Perry R (2018). Health benefits of electrically-assisted cycling: a systematic review. Int J Behav Nutr Phys Act.

[CR8] Byambasukh O, Vinke P, Kromhout D (2021). Physical activity and 4-year changes in body weight in 52,498 non-obese people: the Lifelines cohort. Int J Behav Nutr Phys Act.

[CR9] Chia CW, Egan JM, Ferrucci L (2018). Age-related changes in glucose metabolism, hyperglycemia, and cardiovascular risk. Circ Res.

[CR10] Chrzanowski J, Grabia S, Michalak A (2023). GlyCulator 3.0: a fast, easy-to-use analytical tool for CGM data analysis, aggregation, center benchmarking, and data sharing. Diabetes Care.

[CR11] Cornelissen VA, Smart NA (2013). Exercise training for blood pressure: a systematic review and meta-analysis. J Am Heart Assoc Cardiovasc Cerebrovasc Dis.

[CR12] Currie KD, Thomas SG, Goodman JM (2009). Effects of short-term endurance exercise training on vascular function in young males. Eur J Appl Physiol.

[CR13] Dow CA, Stauffer BL, Brunjes DL (2017). Regular aerobic exercise reduces endothelin-1-mediated vasoconstrictor tone in overweight and obese adults. Exp Physiol.

[CR43] DuBose SN, Li Z, Sherr JL, Beck RW, Tamborlane WV, Shaw VN (2021). Effect of exercise and meals on continuous glucose monitor data in healthy individuals without diabetes. J Diabetes Sci Technol.

[CR14] Fagard RH (2005). Effects of exercise, diet and their combination on blood pressure. J Hum Hypertens.

[CR15] Fernberg U, Fernstrom M, Hurtig-Wennlof A (2021). Higher total physical activity is associated with lower arterial stiffness in Swedish, young adults: the cross-sectional lifestyle, biomarkers, and artherosclerosis study. Vascular Health and Risk Managmt.

[CR16] Golding LA (2000). YMCA fitness testing and assessment manual.

[CR17] Gutch M, Kumar S, Razi SM (2015). Assessment of insulin sensitivity/resistance. Indian J Endocrinol Metab.

[CR18] Guthold R, Stevens GA, Riley LM, Bull FC (2018). Worldwide trends in insufficient physical activity from 2001 to 2016: a pooled analysis of 358 population-based surveys with 1·9 million participants. Lancet Glob Health.

[CR19] Kirwan JP, Barkoukis H, Brooks LM (2009). Exercise training and dietary glycemic load may have synergistic effects on insulin resistance in older obese adults. Ann Nutr Metab.

[CR20] Lakicevic N, Gentile A, Mehrabi S (2020). Make fitness fun: could novelty be the key determinant for physical activity adherence?. Front Psychol.

[CR21] Lewczuk P, Sohnchen N, Kele H (2003). Endothelin-1 concentration in plasma is increased after jogging but decreased after cycling in healthy men. Clin Exp Med.

[CR22] Li XY, Zhao S, Fan XH (2020). Plasma big endothelin-1 is an effective predictor for ventricular arrythmias and end-stage events in primary prevention implantable cardioverter-defibrillator indication patients. J Geriatr Cardiol.

[CR23] Liguori G, Feito Y (2021). ACSM’s guidelines for exercise testing and prescription.

[CR24] Maeda S, Miyauchi T, Lemitsu M (2004). Resistance exercise training reduces plasma endothelin-1 concentration in healthy young humans. J Cardiovasc Pharmacol.

[CR25] McVicar J, Keske MA, Daryabeygi-Khotbehsara R, Betik AC, Parker L, Maddison R (2022). Systematic review and meta-analysis evaluating the effects electric bikes have on physiological parameters. Scand J Med Sci Sports.

[CR26] Mikus CR, Oberlin DJ, Libla J (2012). Glycaemic control is improved by 7 days of aerobic exercise training in patients with type 2 diabetes. Diabetologia.

[CR27] Montoye AHK, Vondrasek JD, Neph SE (2022). Comparison of the activPAL CREA and VANE algorithms for characterization of posture and activity in free-living adults. J Meas Phys Behav.

[CR28] Munan M, Oliveira CLP, Marcotte-Chénard A (2020). Acute and chronic 3ffects of 3xercise on dontinuous glucose monitoring outcomes in Type 2 Diabetes: a meta-analysis. Front Endocrinol (lausanne).

[CR29] Najafipour F, Mobasseri M, Yavari A (2017). Effect of regular exercise training on changes in HbA1c, BMI and VO2max among patients with type 2 diabetes mellitus: an 8-year trial. BMJ Open Diabetes Res Care.

[CR30] Otsuki T, Maeda S, Iemitsu M (2007). Relationship between arterial stiffness and athletic training programs in young adult men. Am J Hypert.

[CR31] Peterman JE, Morris KL, Kram R, Byrnes WC (2016). Pedelecs as a physically active transportation mode. Eur J Appl Physiol.

[CR32] R Core Team (2020). R: A language and environment for statistical computing. R Foundation for Statistical Computing, Vienna, Austria. https://www.R-project.org/

[CR33] Roberts CK, Vaziri ND, Barnard RJ (2002). Effect of diet and exercise intervention on blood pressure, insulin, oxidative stress, and nitric oxide availability. Circulation.

[CR34] Ruegsegger GN, Booth FW (2018). Health benefits of exercise. Cold Spring Harb Perspect Med.

[CR35] Saz-Lara A, Cavero-Redondo I, Álvarez-Bueno C, Notario-Pacheco B, Ruiz-Grao MC, Martínez-Vizcaíno V (2021). The acute effect of exercise on arterial stiffness in healthy subjects: a meta-analysis. J Clin Med.

[CR36] Shah VN, DuBose SN, Li Z (2019). Continuous glucose monitoring profiles in healthy nondiabetic participants: a multicenter prospective study. J Clin Endocrinol Metab.

[CR37] Smith JAB, Savikj M, Sethi P (2021). Three weeks of interrupting sitting lowers fasting glucose and glycemic variability, but not glucose tolerance, in free-living women and men with obesity. Am J Physiol-Endocrinol Metab.

[CR38] Stamatelopoulos K, Tsoltos N, Armenia E (2020). Physical activity is associated with lower arterial stiffness in normal-weight postmenopausal women. J Clin Hypertens.

[CR40] Warburton DER, Bredin SSD (2017). Health benefits of physical activity: a systematic review of current systematic reviews. Curr Opin Cardiol.

[CR41] Whitfield G (2020) Monitoring state-level changes in walking, biking, and taking public transit to work—American Community Survey, 2006 and 2017. https://www.cdc.gov/pcd/issues/2020/20_0097.htm. Accessed 13 Mar 2023 10.5888/pcd17.200097 PMC7553222 33006545

[CR42] Withall J, Jago R, Fox KR (2011). Why some do but most don’t Barriers and enablers to engaging low-income groups in physical activity programmes: a mixed methods study. BMC Public Health.

